# Traditional Chinese medicine (TCM) collaborative care for patients with axial spondyloarthritis (AcuSpA): protocol for a pragmatic randomized controlled trial

**DOI:** 10.1186/s13063-018-3117-2

**Published:** 2019-01-14

**Authors:** Yu Heng Kwan, Warren Fong, Xiang Ling Ang, Chuen Seng Tan, Bee Choo Tai, Youyi Huang, Marcel Bilger, Jie Kie Phang, Hui Chin Tan, Jia Ven Lee, Limin Sun, Choy Tip Tan, Bao Qiang Dong, Hwee Ling Koh, Ying Ying Leung, Nai Lee Lui, Siaw Ing Yeo, Swee Cheng Ng, Kok Yong Fong, Julian Thumboo, Truls Østbye

**Affiliations:** 1 Program in Health Services and Systems Research, Duke-NUS Medical School, Singapore, Singapore; 2 Department of Rheumatology and Immunology, Singapore General Hospital, Academia Building, Level 4, 20 College Road, Singapore, 169856 Singapore; 3 Duke-NUS Medical School, Singapore, Singapore; 4 Department of Medicine, Yong Loo Lin School of Medicine, National University of Singapore, Singapore, Singapore; 5 Singapore Thong Chai Medical Institution, Singapore, Singapore; 6 Saw Swee Hock School of Public Health, National University of Singapore, Singapore, Singapore; 7 Internal Medicine Residency, SingHealth, Singapore, Singapore; 8 Liaoning University of Traditional Chinese Medicine, Shenyang, Liaoning People’s Republic of China; 9 Department of Pharmacy, National University of Singapore, Singapore, Singapore

## Abstract

**Background:**

Axial spondyloarthritis (AxSpA) is a chronic disease which results in fatigue, pain, and reduced quality of life (QoL). Traditional Chinese medicine (TCM), especially acupuncture, has shown promise in managing pain. Although a TCM collaborative model of care (TCMCMC) has been studied in cancer, there are no randomized controlled trials investigating TCM in AxSpA. Therefore, we will conduct a pragmatic trial to determine the clinical effectiveness, safety, and cost-effectiveness of TCMCMC for patients with AxSpA. We define TCMCMC as standard TCM history taking and physical examination, acupuncture, and TCM non-pharmacological advice and communications with rheumatologists in addition to usual rheumatologic care. The purpose of this paper is to describe the rationale for and methodology of this trial.

**Methods/design:**

This pragmatic randomized controlled trial will recruit 160 patients who are diagnosed with AxSpA and have inadequate response to non-steroidal anti-inflammatory drugs (NSAIDs). Simple randomization to usual rheumatologic care or the intervention (TCMCMC) with a 1:1 allocation ratio will be used. Ten 30-min acupuncture sessions will be provided to patients assigned to the TCMCMC arm. All participants will continue to receive usual rheumatologic care. The primary endpoint — spinal pain — will be evaluated at week 6. Secondary endpoints include clinical, quality of life, and economic outcome measures. Patients will be followed up for up to 52 weeks, and adverse events will be documented.

**Discussion:**

This trial may provide evidence regarding the clinical effectiveness, safety, and cost-effectiveness of a TCMCMC for patients with AxSpA.

**Trial registration:**

ClinicalTrials.gov, NCT03420404. Registered on 14 February 2018.

**Electronic supplementary material:**

The online version of this article (10.1186/s13063-018-3117-2) contains supplementary material, which is available to authorized users.

## Background

Axial spondyloarthritis (AxSpA) is a chronic debilitating disease, often affecting the quality of life of patients [1, 2]. There is no cure for AxSpA, and the pathophysiology of the disease remains unclear [3, 4]. The recommended treatment for patients with AxSpA who remain symptomatic after initial treatment with non-steroidal anti-inflammatory drugs (NSAIDs) usually involves biologics which cost more than USD$20,000 per year [5]. Biologics may also have significant side effects, in particular, increased incidence of infections such as tuberculosis and risk of malignancies [6, 7]. There is thus a need for alternative treatment for patients who have inadequate response to NSAIDs but cannot be on biologics due to side effects or financial reasons.

Traditional Chinese medicine (TCM) is one of the most commonly used complementary and alternative medicine modalities [8–10]. TCM, especially acupuncture, has shown promising results in the management of pain, possibly by releasing encephalin [11–13]. Acupuncture has frequently been promoted for lower back pain and osteoarthritis [14], and rheumatic diseases are, according to survey data, frequently treated by acupuncturists [15–17]. Previous studies have demonstrated the efficacy of acupuncture in pain relief for various rheumatic diseases [18], with minimal side effects [19]. For patients with irritable bowel syndrome, acupuncture plus usual care can provide additional benefit over usual care alone, and the magnitude of the effect is sustained [20]. Hence, acupuncture may be a safe and effective intervention to relieve pain.

**Fig. 1:**
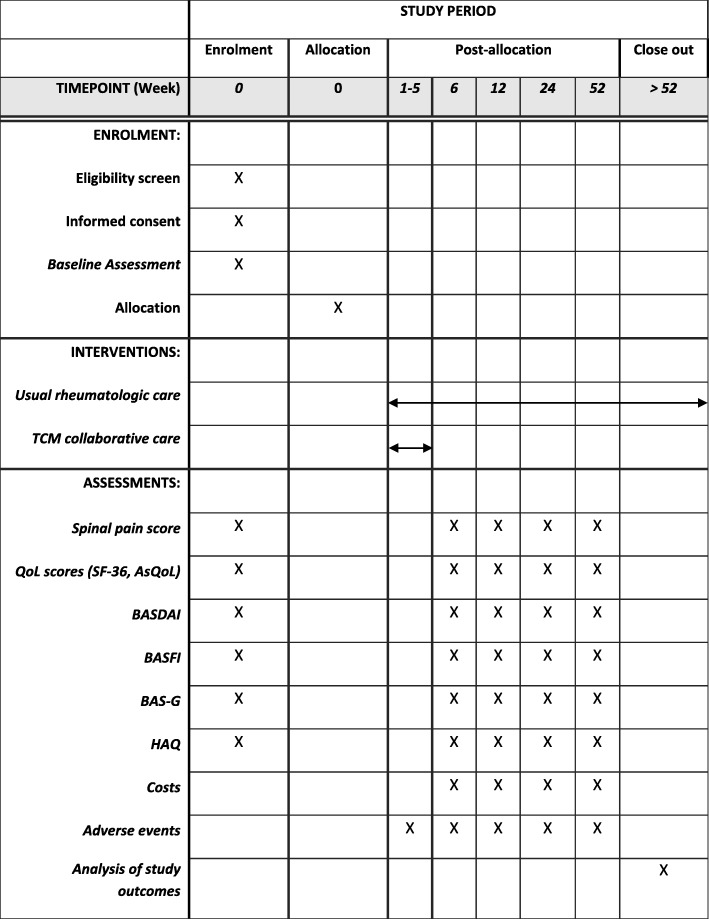
SPIRIT figure for the schedule of enrollment, interventions, and assessments. Abbreviations: *ASQoL* Ankylosing Spondylitis Quality of Life, *BASDAI* Bath Ankylosing Spondylitis Disease Activity Index, *BASFI* Bath Ankylosing Spondylitis Functional Index, *BAS-G* Bath Ankylosing Spondylitis Global score, *HAQ* Health Assessment Questionnaire, *QoL* quality of life, *SF-36* 36-item Short Form Health Survey, *SPIRIT* Standard Protocol Items: Recommendations for Interventional Trials, *TCM* traditional Chinese medicine

There has been no randomized controlled trial to assess the effectiveness of TCM, in addition to usual care, for patients with AxSpA [19]. Given this evidence gap and the unmet need of the patients with AxSpA who do not respond well to current conventional treatment, further investigations of a collaborative model of care involving TCM are merited. Hence, we will assess the clinical effectiveness, safety, and cost-effectiveness of a TCM collaborative model of care (TCMCMC) in patients with AxSpA using a pragmatic trial approach. In this study, we aim to provide a TCMCMC as closely as possible to how it would be provided in the real world. This design can provide evidence of effectiveness, which may be important for policy- and decision-makers considering TCM as a treatment option for patients with AxSpA. In this manuscript, we describe the rationale and the detailed methodology of the trial. This protocol is guided by the Standard Protocol Items: Recommendations for Interventional Trials (SPIRIT) (Fig. 1 and Additional file 1: Table S1).

## Methods/design

### Study design and setting

**Fig. 2:**
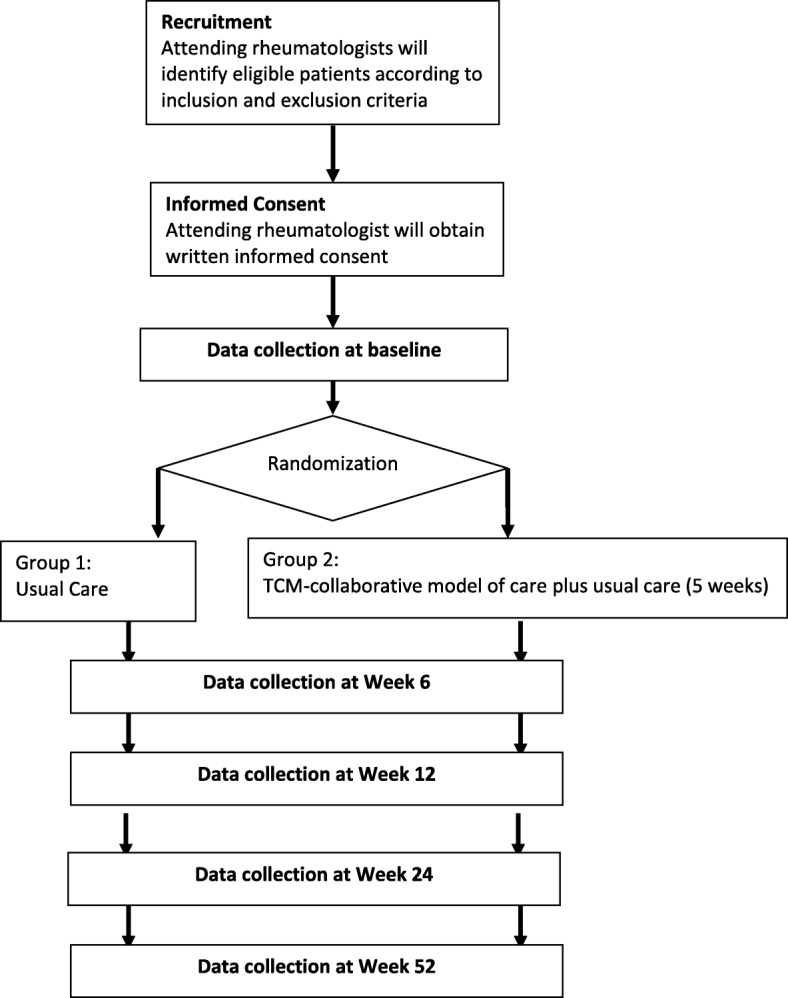
Trial work plan. Follow-up will be performed at weeks 6, 12, 24, and 52 after the baseline visit. Pain score at week 6 is the primary outcome measure for this study. Outcome measures at week 52 are exploratory. The remaining outcome measures are the secondary outcomes of the study

This is a two-arm, pragmatic, randomized controlled trial to evaluate the clinical effectiveness, safety, and cost-effectiveness of TCMCMC, in particular acupuncture, for patients with AxSpA with inadequate response to NSAIDs [21–24]. It is anchored in the Pragmatic Explanatory Continuum Indicator Summary Framework-2 (PRECIS-2) criteria and the extended Consolidated Standards of Reporting Trials (CONSORT) guidelines for pragmatic trials as well as the Standards for Reporting Interventions in Clinical Trials of Acupuncture (STRICTA) statement for acupuncture. The two arms are (1) TCMCMC (including acupuncture) plus usual rheumatologic care and (2) usual rheumatologic care alone. Participants will be recruited from dedicated clinics in a tertiary hospital setting. Recruitment is mainly through doctors’ referrals and recruitment posters and brochures. Patients will be randomly allocated to receive usual rheumatologic care or the intervention (TCMCMC) with a 1:1 allocation ratio via random permuted block randomization. The trial work plan is summarized in Fig. 2. This paper is based on protocol version 3.0, 30 August 2018.

### Inclusion/exclusion criteria

We aim to recruit patients with AxSpA who have spinal pain and active disease despite standard medical therapy. Patients are eligible for the study if they are 21 years of age or older; have AxSpA, diagnosed according to the 2009 Assessment of Spondyloarthritis International Society (ASAS) criteria [25]; have active disease based on Bath Ankylosing Spondylitis Disease Activity Index (BASDAI) score ≥ 4 on a 11-point numerical rating sScale (NRS) and spinal pain score ≥ 4 on a 11-point NRS [26]; have failed two sequential NSAIDs (including cyclooxygenase-2 inhibitor) at maximal tolerated doses for ≥4 weeks in total; and have received no biologic therapy (i.e., tumor necrosis factor blocker or anti-interleukin 17) within the past 3 months. Patients who are on current treatment with concomitant methotrexate (MTX) or sulfasalazine (SSZ) at study entry must be on the drug for ≥ 12 weeks and at stable dose for ≥ 4 weeks prior to randomization. Patients who are on non-biologic disease-modifying antirheumatic drugs (DMARDs) other than MTX or SSZ must discontinue the DMARD 4 weeks prior to randomization, except for leflunomide, which has to be discontinued for 8 weeks prior to randomization unless a cholestyramine washout has been performed. Patients taking systemic corticosteroids have to be on a stable dose of ≤10 mg/day prednisolone or equivalent for at least 2 weeks before randomization. Patients with a BASDAI 50% response to NSAIDs will be recruited in one block, while patients who did not have a BASDAI 50% response to NSAIDs will be recruited in another block.

We will exclude female patients who are pregnant or breastfeeding; on antiplatelet agents (i.e., aspirin, clopidogrel, dipyridamole, etc.) or anticoagulants (i.e., warfarin, enoxaparin, rivaroxaban, dabigatran, etc.); have bleeding disorders; or have blood-borne communicable diseases (e.g., hepatitis B, hepatitis C, human immunodeficiency virus, etc.).

### Blinding

The attending rheumatologist will identify eligible patients according to the inclusion and exclusion criteria. Informed consent will be taken by the attending rheumatologist, and participants will be referred to a research coordinator who will randomly assign them to the intervention or control arm. The research coordinator will have custody of the randomization list that was pre-generated by the biostatistician using a computerized random number generator, and will assign treatment accordingly. The attending rheumatologist who will enroll participants will not be aware of the allocation sequence.

The randomization list is kept by the biostatistician and research coordinator until the end of the study to ensure allocation concealment; therefore, the data analysts will be kept blinded to the allocation. The participants will be instructed not to disclose the allocation to the attending rheumatologist.

### Control group

Usual rheumatologic care, guided by the management guidelines developed by ASAS, consists of a referral to the physiotherapist for therapeutic exercise, medications including NSAIDs, and regular monitoring for complications which may arise from AxSpA such as uveitis, interstitial lung disease, and risk factors for cardiovascular disease and osteoporosis [27]. In this trial, the attending rheumatologist will see each patient at regular intervals (ranging from 6 weeks to 6 months) depending on the patient’s condition as per routine care. At each session, the attending rheumatologist will conduct a thorough physical examination and monitor the disease activity using validated patient-reported outcome measures. The rheumatologist will be allowed to prescribe the full range of medications, including biological agents, as per routine care and according to local treatment guidelines. There are no concomitant or prohibited interventions during this trial. The rheumatologist will remind patients not to visit any TCM physician and not to seek alternative therapy for the duration of the study.

### Intervention group

TCM physicians registered with the Singapore Traditional Chinese Medicine Practitioners Board with at least 3 years of experience will participate in the study. Prior to the treatment sessions, all the acupuncturists will undergo training to ensure standardization of acupuncture techniques. The intervention arm will involve the TCM physicians in the management of the patients, in addition to usual rheumatologic care. The clinical interventions were designed and agreed on by senior TCM physicians in consultation with rheumatologists. The clinical interventions to be carried out by the TCM physicians include counseling, diagnosis based on TCM clinical syndromes, and the prescription of acupuncture.

**Fig. 3:**
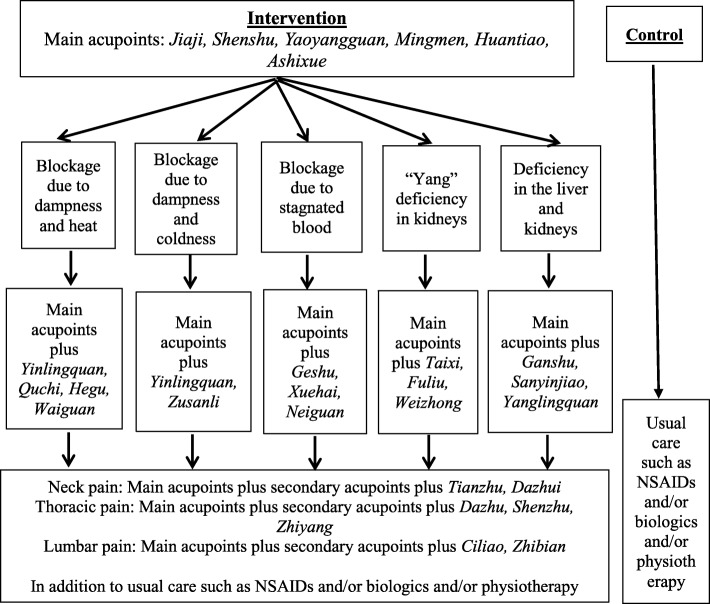
The acupuncture points employed in this study. The main acupuncture points are *Jiaji*, *Shenshu*, *Yaoyangguan*, *Mingmen*, *Huantiao*, *Ashixue*. The patients in the intervention group will be classified into one of the five syndromes based on their clinical presentation and have secondary acupuncture points chosen based on their respective syndromes. There will also be additional acupuncture points for patients with neck pain, thoracic pain, and/or lumbar pain. Patients in both the intervention and control groups will be given usual care consisting of NSAIDs and/or biologics and/or physiotherapy as deemed necessary by the attending rheumatologists. Abbreviations: *Acupoints* acupuncture points, *NSAIDs* non-steroidal anti-inflammatory drugs

The main acupuncture points of interest have been identified a priori as *Jiaji*, *Shenshu*, *Yaoyangguan*, *Mingmen*, *Huantiao*, and *Ashixue*. We will use sterile disposable stainless-steel needles of 0.25 mm diameter, 25 mm or 40 mm long and 0.30 mm diameter, 50 mm or 70 mm long, depending on the acupuncture points. In addition to the main acupuncture points specific for the treatment of AxSpA, the TCM physicians will be allowed to make minor adjustments to the acupuncture points in view of the differing constitution of the patients as per the holistic treatment philosophy of TCM as shown in Fig. 3. Therefore, the number of needles to be inserted per subject per session will differ. The needles will be inserted from 0.25 to 1 in. deep depending on the acupuncture points. After eliciting the *de qi* response, the needles will be left in place for 30 min. The needles will be stimulated manually every 10 min. The acupuncture treatment will consist of a total of 10 sessions (or 2 courses) in total. Each course of treatment will consist of 5 acupuncture sessions held over 2 weeks, each session lasting 30 min. The patient will have a break of at least 3 days and up to 1 week in between each course of acupuncture. TCM physicians will document components of treatment and adherence in standardized logbooks. The reporting of the intervention is guided by STRICTA guidelines (Additional file 1: Table S2).

### Primary outcome

The outcomes selection was guided by the ASAS and Outcome Measures in Rheumatology (OMERACT) core domains for SpA [27]. Spinal pain score at week 6 is the primary outcome of this study, since pain is what matters most to patients with AxSpA. The 11-point pain NRS (range 0–10), which is self-administered and validated across many settings, will be used [28]. The 6-week time-point was selected to investigate if there is short-term efficacy in the intervention. The primary and secondary outcome measures are shown in Table 1.

**Table 1:** Summary of primary, secondary, and exploratory outcome measures

Outcomes	Definition	Time			
		Week 6	Week 12	Week 24	Week 52
1. Primary outcome					
1.1 Spinal pain score	Overall level of pain at neck, back, or hip	✓			
2. Main secondary outcome					
2.1 Spinal pain score	Overall level of pain at neck, back, or hip			✓	
3. Other secondary outcomes					
3.1 Clinical outcomes					
3.1.1 Spinal pain score	Overall level of pain at neck, back, or hip		✓		
3.1.2 BASDAI	Disease activity of patient	✓	✓	✓	
3.1.3 BASFI	Disease-specific physical function	✓	✓	✓	
3.1.4 BAS-G	Global assessment of disease	✓	✓	✓	
3.1.5 HAQ	Disability	✓	✓	✓	
3.2 Quality of life outcomes					
3.2.1 SF-36	General QoL assessment	✓	✓	✓	
3.2.2 ASQoL	Disease-specific QoL assessment	✓	✓	✓	
3.3 Economic outcomes					
3.3.1 Costs	Direct and indirect costs of disease	✓	✓	✓	
4. Exploratory outcomes					
4.1 Clinical outcomes					
4.1.1 Spinal pain score	Overall level of pain at neck, back, or hip				✓
4.1.2 BASDAI	Disease activity of patient				✓
4.1.3 BASFI	Disease-specific physical function				✓
4.1.4 BAS-G	Global assessment of disease				✓
4.1.5 HAQ	Disability				✓
4.2 Quality of life outcomes					
4.2.1 SF-36	General QoL assessment				✓
4.2.2 ASQoL	Disease-specific QoL assessment				✓
4.3 Economic outcomes					
4.3.1 Costs	Direct and indirect costs of disease				✓

Baseline data will be collected for all outcome measures except for costs

Abbreviations: *ASQoL* Ankylosing Spondylitis Quality of Life, *BASDAI* Bath Ankylosing Spondylitis Disease Activity Index, *BASFI* Bath Ankylosing Spondylitis Functional Index, *BAS-G* Bath Ankylosing Spondylitis Global score, *HAQ* Health Assessment Questionnaire, *SF-36* 36-item Short Form Health Survey, *QoL* quality of life

### Main secondary outcome

Spinal pain score at week 24 is the main secondary outcome in this study. This time-point was selected to investigate if there is sustained long-term effectiveness for the intervention.

### Other secondary outcomes

We will also collect secondary clinical outcomes, quality of life outcomes, and economic outcomes. Secondary clinical outcomes include spinal pain at week 12 and week 24 and clinical parameters, including BASDAI [29], Bath Ankylosing Spondylitis Functional Index (BASFI) [30], Bath Ankylosing Spondylitis Global score (BAS-G) [31], and Health Assessment Questionnaire (HAQ) [32, 33] at weeks 6, 12, and 24.

We will assess quality of life (QoL) using the 36-item Short Form Health Survey (SF-36) [34] and the Ankylosing Spondylitis Quality of Life (ASQoL) questionnaire [35, 36], which will be administered at baseline and at weeks 6, 12, and 24. Both patient-reported outcomes have been validated in the Singapore population [34].

*For secondary economic outcomes*, AxSpA-related and non-AxSpa-related healthcare use such as rheumatologist’s consultation fees, costs of laboratory procedures, and number of inpatient days will be collected. Healthcare use will be obtained through questionnaires administered to patients in both the control and intervention arms at weeks 6, 12, and 24. In addition, this information will be supplemented by medical records and data from the electronic databases. Non-healthcare financial consequences will be captured in the questionnaires by recording self-reported travel costs incurred by the patients to receive treatment, patient income and salary, the number of days patients missed work due to illness, and work status (active, inactive, retired).

### Exploratory outcomes

The spinal pain, BASDAI, BASFI, BAS-G, HAQ, SF-36, and ASQoL results, rheumatologist’s consultation fees, cost of laboratory procedures, and number of inpatient days at week 52 will serve as exploratory outcomes.

### Instruments and definitions

BASDAI (ranging from 0 to 10) is an English, self-administered, disease-specific questionnaire to measure disease activity, with higher values indicating more active disease [37]. BASFI (ranging from 0 to 10) is a disease-specific questionnaire used to measure physical functioning, with higher values indicating worse functioning [37]. BAS-G (ranging from 0 to 100) is a disease-specific questionnaire to give a global assessment of well-being, with higher score reflecting poorer well-being [31].

HAQ (ranging from 0 to 3) is a self-administered, generic questionnaire used to assess disability, with a higher score reflecting worse disability [38]. It includes eight domains: dressing, arising, eating, walking, hygiene, reach, grip, and daily activities. The English version of HAQ is a valid and reliable measure for use in patients with AxSpA in Singapore [33].

SF-36 version 2 is a self-administered, generic questionnaire used to measure QoL in eight areas of perceived health. The 36 individual questions make up 8 subscales, with lower scores reflecting poorer QoL. Norm-based scores were used whereby 50 represents the mean and 10 represents the standard deviation. The eight subscales are physical functioning, role limitation due to physical problems, bodily pain, general health, vitality, social functioning, and role limitation due to emotional problem and mental health. These eight subscales were summed with different weights to give two summary scores: physical component summary (PCS) and mental component summary (MCS) [39]. The English version of SF-36 has been shown to be valid and reliable in patients with AxSpA in Singapore [34].

ASQoL is a self-administered, patient-derived, and disease-specific measure of QoL for AS. It consists of 18 items with a “yes” (scored as 1) or “no” (scored as 0) response to each item. All item scores are summed to a total score ranging from 0 to 18 [40], with higher scores indicating worse QoL. The English version of ASQoL has been shown to be a valid and reliable measure for use in patients with AxSpA, also in Singapore [36].

### Process measures

Adherence to treatment defined as the number of acupuncture sessions attended by the patients in the intervention arm will be assessed. We will also record the number of acupuncture and rheumatology consultations and the number of needles used in each acupuncture session. The TCM physicians will complete a checklist to ensure standardization of acupuncture treatment across all sessions (Additional file 1: Table S3).

### Safety

For this study, an adverse event (AE) is any untoward medical occurrence in a patient which does not necessarily have a causal relationship with the treatment. An AE can therefore be any unfavorable and unintended sign (including an abnormal laboratory finding), symptom, or disease temporally associated with the treatment. A serious adverse event (SAE) is any untoward medical occurrence that at any dose results in death, is life-threatening, requires inpatient hospitalization or prolongation of existing hospitalization, results in persistent or significant disability/incapacity, or is a congenital anomaly/birth defect. However, pre-planned hospitalizations before recruitment will not be recorded as AEs.

AEs will be recorded throughout the study up to week 52. Acupuncture-related AEs are defined as symptoms or complications related to acupuncture that begin or worsen after the first session of acupuncture through the last session of acupuncture [41]. We adapted the AEs of interest from a systematic review of AEs of acupuncture treatment, including broken needles, fainting during a session, and local infections at the site of acupuncture.

AEs related to usual rheumatologic care were adapted from previously conducted randomized controlled trials performed in patients with AxSpA [42, 43]. Adverse effects related to usual rheumatologic care include abdominal distension and pain, nausea, diarrhea, gastrointestinal disease, headache, giddiness, upper respiratory tract infection, nasopharyngitis, malignancy, infections, and hepatic-related AEs. These AEs will be recorded.

### Sample size justification

As this is the first study to explore a collaborative model between TCM physician and rheumatologists in the field of AxSpA, we based our sample size calculation on a study by Meng et al. [11]. With a conservative estimate of 0.6 point difference on a 11-point scale in spinal pain score between the two arms, and assuming a standard deviation of 1.2 for both arms, approximately 64 patients are needed for each arm to obtain a statistical power of 80% (two-sided type I error rate of 0.05) based on a 1:1 treatment allocation. Allowing for a dropout rate of 20%, a total of 160 patients with 80 patients per arm will be needed. According to Cohen, this effect size is considered “moderate” [44].

### Data collection and management

To ensure the accuracy of outcome assessments and data collection, the investigators and research coordinator will attend a training workshop before the start of the trial. All attendees will be provided with a protocol and will discuss the topics they may feel confused about until everyone is totally clear about the procedures. Data will be entered into SingHealth Research Electronic Data Capture (REDCap) [45], which is a password-protected software application designed to collect and manage data for research studies. All hardcopy documents will be kept in a locked cabinet. Range checks will be implemented for data values in REDCap to promote data quality. There is no plan to promote participant retention and complete follow-up.

A data and safety monitoring board, independent from the sponsor and competing interests, will meet annually to provide interim monitoring of the safety and efficiency data for the study. The data and safety monitoring board, comprising senior clinical experts and external biostatisticians, will help ensure the availability of appropriate expertise in trial design, execution, interim monitoring, analysis, and reporting. After each interim analysis, the data and safety monitoring board will determine whether it is necessary to continue, modify, or terminate the collection of these outcome data.

### Statistical analysis

The statistical analysis will be performed on an intention-to-treat (ITT) basis. The primary outcome of interest is the difference in pain score at week 6 between the intervention and control arms. The baseline characteristics will be shown as the mean ± standard deviation (or median and interquartile range where adequate) for continuous variables (e.g., age) and *n* (%) for categorical variables (e.g., gender). Primary outcome of pain score at week 6 will be compared between the arms using Student’s *t* test. Further adjustment will be made with baseline pain score using analysis of covariance (ANCOVA). Multiple imputations will be used to account for missing values. All evaluations will be made assuming a two-sided type I error rate set at 0.05.

For secondary outcomes with repeated measurements (at weeks 6, 12, 24, and 52), we will use a linear mixed model to account for within-individual correlation among measurements and the sandwich estimator to obtain robust standard error estimates. The intervention indicator and time factor will be included among the linear predictors adjusting for baseline covariates.

To assess the safety of the intervention, we will present the frequency of AEs that occurred, expressed in frequency and percentages, in both the intervention and control arms within the study period of 52 weeks.

The economic evaluation will be conducted from both the healthcare system and societal perspectives. Both a cost-effectiveness analysis (i.e., cost of reduction in 1 pain score point) and cost-utility analysis (i.e., cost of reduction in 1 quality-adjusted life year saved) will be performed [46, 47]. Costs will include direct healthcare-related costs of the TCM physician, rheumatologist, hospital stays, and any drugs. The indirect costs caused by lost workdays will also be considered. Health utility will be measured using the Short-Form Six-Dimension (SF-6D) scale, which is a derivation of the SF-36 scale [48]. The cost-effectiveness and cost-utility analyses will be performed by calculating the incremental cost-effectiveness ratio and incremental cost-utility ratio respectively. The analysis period will be at weeks 6, 12, 24, and 52.

The incremental cost-effectiveness ratio will be calculated by dividing the between-group difference in costs by the between-group difference in effects (i.e., costs per pain score reduced). The incremental cost-utility ratio will be calculated by dividing the between-group difference in costs by the between-group difference in utility (i.e., costs per SF-6D unit improvement). Sensitivity analysis will be conducted on the most important cost drivers to assess the robustness of the results.

One interim analysis will be performed by the trial statistician. The statistician will report to the independent data and safety monitoring board, which will provide recommendations without revealing any detailed results on treatment effect. The principal investigator then decides on the continuation of the trial and will report to the ethics committee. There is no formal stopping rule for this study.

## Ethics and dissemination

This study has been approved by the SingHealth Centralized Institutional Board Review (CIRB) (Reference number 2017/2088). Independent clinicians and biostatisticians with extensive research experience in clinical trials will serve as the Data and Safety Monitoring Committee. The SingHealth Office of Research Integrity and Compliance (ORIC) may perform random audits to ensure that relevant regulations and guidelines are met. Study participation is voluntary and can be discontinued at any time, and deciding not to take part will not affect a patient’s care. Protocol amendments, adverse effects reporting, and annual review will be overseen by the CIRB. The information provided by the patients will only be shared with members of the research team. Every effort will be made to keep patient information confidential. All personal identifying information and research data will be stored on SingHealth REDCap, which is a password-protected network. All research-related paper documents will be kept in a locked cabinet. All patient information will be kept strictly confidential. All members of the research team are required to complete a biomedical research training module offered by the Collaborative Institutional Training Initiative (CITI) on human subjects’ protection and data security.

The hospital does not make any provisions to compensate study participants for research-related injury. However, compensation may be considered on a case-by-case basis for unexpected injuries due to non-negligent causes. These costs will be covered using the blanket insurance for clinical trials (Ministry of Health Clinical Trial Insurance) conducted in SingHealth.

The results of this study will be disseminated by presentation at international conferences and publication in peer-reviewed journals. All investigators involved with this study will have access to the final trial dataset. Participants will be informed about the results of the study.

## Discussion

This is the first study to assess the impact of involving TCM, in particular acupuncture, in the management of patients with AxSpA with inadequate response to NSAIDs. By utilizing a pragmatic trial approach, we aim to estimate the effect of this intervention in the real-world setting. Pursuant to the PRECIS-2, our design reflects key pragmatic dimensions: (1) we will recruit patients who are most likely to use TCM (i.e., patients who have inadequate disease control despite NSAIDs and declined or are unable to afford biologics); (2) the treatment setting reflects the situation where the patients will receive their treatment in real life (3); extra resources are not provided for the treatment, and this reflects what would be done currently in clinical practice (4); relevant outcomes which are important to the patients and stakeholders in healthcare are chosen (5); no extra follow-up is scheduled with the patients, but any extra data needed from the data collection will be collected in their home or over the telephone; and (6) we follow an ITT analysis [22, 49]. With these features, our trial balances the issues of internal and external validity, with the goal of assessing real-world effectiveness of a TCM collaborative model of care involving both a TCM physician and a rheumatologist in the management of patients with AxSpA with inadequate response to NSAIDs [50].

For AxSpA, NSAIDs are often the first-line treatment, and biologics are the step-up treatment for patients who have inadequate response to NSAIDs [27]. However, the cost difference between NSAIDs and biologics is very large [51]. Patients who have inadequate response to NSAIDs but cannot afford biologics often experience significant pain and impairment in QoL [52]. Hence, our trial will provide evidence for a novel model of care for this group of patients.

In conclusion, a pragmatic trial of a TCM collaborative model of care involving both a TCM physician and rheumatologist in the management of patients with AxSpA may provide evidence to support the referral of patients with AxSpA to and collaboration with TCM physicians for better management of pain and QoL. This may aid policy- and decision-makers considering TCM physicians as a referral option for patients with AxSpA and as collaborators for allopathic primary care physicians and rheumatologists.

## Trial status

At the time of manuscript submission, recruitment for the study is underway but not completed.

## Additional file

Additional file 1: **Table S1.** SPIRIT 2013 checklist: recommended items to address in a clinical trial protocol and related documents. **Table S2.** Checklist for items in STRICTA 2010. **Table S3.** Checklist to ensure standardization of acupuncture treatment. (DOCX 37 kb)

## Abbreviations

ANCOVA: Analysis of covariance
ASAS: Assessment of Spondyloarthritis International Society
ASQoL: Ankylosing Spondylitis Quality of Life
AxSpA: Axial spondyloarthritis
BASDAI: Bath Ankylosing Spondylitis Disease Activity Index
BASFI: Bath Ankylosing Spondylitis Functional Index
BAS-G: Bath Ankylosing Spondylitis Global score
CONSORT: Consolidated Standards of Reporting Trials
DMARD: Disease-modifying antirheumatic drug
ITT: Intention to treat
MTX: Methotrexate
NRS: Numerical rating scale
NSAID: Non-steroidal anti-inflammatory drug
OMERACT: Outcome Measures in Rheumatology
PRECIS-2: Pragmatic Explanatory Continuum Indicator Summary Framework-2
QOL: Quality of life
REDCap: Research Electronic Data Capture
SF-36: 36-item Short Form Health Survey
SF6D: Short-Form Six-Dimension
SPIRIT: Standard Protocol Items: Recommendations for Interventional Trials
SSZ: Sulfasalazine
STRICTA: Standards for Reporting Interventions in Clinical Trials of Acupuncture
TCM: Traditional Chinese medicine
TCMCMC: Traditional Chinese medicine collaborative model of care
